# 含苯达莫司汀预处理方案自体造血干细胞移植治疗淋巴瘤的近期疗效及安全性

**DOI:** 10.3760/cma.j.issn.0253-2727.2022.01.012

**Published:** 2022-01

**Authors:** 立才 安, 英慧 刘, 婧瑶 王, 俊杰 马, 俊卿 徐, 凯敏 李, 荣霞 魏, 晶蕊 隋, 祥艳 冯, 小倩 刘, 丽明 陈, 晓霞 初

**Affiliations:** 烟台毓璜顶医院血液内科，烟台 264000 Department of Hematology, Yantai Yuhuangding Hospital, Yantai 264000, China

自体造血干细胞移植（ASCT）在恶性淋巴瘤整体治疗中具有重要的地位[1]–[2]。常用的预处理方案包括BEAM方案（卡莫司汀、依托泊苷、阿糖胞苷、美法仑）、BEAC方案（卡莫司汀、依托泊苷、阿糖胞苷、环磷酰胺）、CBV方案（环磷酰胺、依托泊苷、卡莫司汀）[3]–[4]。由于卡莫司汀供应短缺，寻找其他替代药物势在必行。苯达莫司汀（bendamustine，Ben）是一种兼具烷化剂和抗代谢作用的双功能氮芥衍生物，在其他烷化剂耐药的情况下，仍具有杀伤肿瘤细胞作用[5]–[6]。本研究对符合ASCT适应证的恶性淋巴瘤患者，给予含Ben方案预处理ASCT，对其近期疗效及安全性进行探索。

## 病例与方法

1. 病例资料：回顾性分析2020年3月至2021年4月在烟台毓璜顶医院血液科接受含Ben方案预处理ASCT的15例恶性淋巴瘤患者。纳入标准：①经活检病理组织学和免疫组织化学以及原位杂交检测符合 2016 WHO淋巴瘤诊断标准[7]；②初治侵袭性外周Ｔ细胞淋巴瘤、中高危及高危弥漫大B细胞淋巴瘤、复发或难治侵袭性淋巴瘤患者。

2. 预处理方案：BeEAM 方案：Ben 150～160 mg/m^2^，静脉滴注，−8 d、−7 d；依托泊苷 100 mg/m^2^，静脉滴注，每12 h 1次，−6 d～−3 d；阿糖胞苷 100 mg/m^2^，静脉滴注，每12 h 1次，−6 d～−3 d；美法仑140 mg/m^2^，−2 d。BeEAC 方案：Ben 150～160 mg/m^2^，静脉滴注，−7 d、−6 d；环磷酰胺1000 mg/m^2^，静脉滴注，−5 d～−2 d；依托泊苷及阿糖胞苷用法同BeEAM方案，−5 d～−2 d。

3. 支持治疗：常规预防出血性膀胱炎、肝静脉闭塞病。所有患者造血干细胞回输后24～48 h予以聚乙二醇化粒细胞集落刺激因子6 mg促进粒系重建。造血干细胞回输后PLT<75×10^9^/L时给予重组人血小板生成素促进巨核系重建。监测血常规，必要时输注滤除白细胞机采血小板和滤除白细胞悬浮红细胞。

4. 造血重建标准：粒细胞植入时间：停用G-CSF后，中性粒细胞绝对计数（ANC）>0.5×10^9^/L连续3 d的第1天；血小板植入时间：当脱离输注血小板时，PLT>20×10^9^/L连续7 d的第1天。

5. 安全性评价：预处理相关不良反应评价根据2017年美国卫生和公共服务部发布的常见不良反应术语评定标准（CTCAE5.0）。移植相关死亡率（TRM）定义为移植后与原发疾病无关的治疗相关死亡率。

6. 疗效评估和随访：疗效评价采用2014年Lugano会议修订标准[8]进行，具体分为完全缓解（CR）、部分缓解（PR）、疾病稳定（SD）、疾病进展（PD）。移植近期疗效评估在回输造血干细胞后3个月进行。

## 结果

1. 临床特征：15例恶性淋巴瘤患者中，男7例，女8例，中位年龄49（18～62）岁。初治患者7例，复发/难治者8例。病理类型、Ann Arbor分期、IPI评分、乳酸脱氢酶水平、是否有大包块、移植前治疗线数和移植前状态详见表1。7例初治患者经诱导化疗达首次CR后接受ASCT，包括中高危及高危弥漫大B细胞淋巴瘤5例，外周T细胞淋巴瘤1例，血管免疫母细胞性T细胞淋巴瘤1例。8例复发/难治患者中4例为经典型霍奇金淋巴瘤，2例为中高危、高危弥漫大B细胞淋巴瘤，1例为滤泡淋巴瘤转化为弥漫大B细胞淋巴瘤，1例为血管免疫母细胞性T细胞淋巴瘤，其中3例ASCT前仍处于SD/PD状态。

**表1:** 15例接受含苯达莫司汀预处理自体造血干细胞移植恶性淋巴瘤患者的临床特征

特征	数值
年龄［岁，*M*（范围）］	49（18~62）
性别［例（％）］	
男	7（46.7）
女	8（53.3）
疾病类型［例（％）］	
霍奇金淋巴瘤	4（26.7）
非霍奇金淋巴瘤	
弥漫大B细胞淋巴瘤	7（46.7）
滤泡性淋巴瘤转化的弥漫大B细胞淋巴瘤	1（6.7）
外周T细胞淋巴瘤	1（6.7）
血管免疫母细胞性T细胞淋巴瘤	2（13.3）
Ann Arbor分期［例（％）］	
Ⅱ期	1（6.7）
Ⅲ期	6（40.0）
Ⅳ期	8（53.3）
IPI评分（仅非霍奇金淋巴瘤）［例（％）］	
中高危	8（72.7）
高危	3（27.3）
乳酸脱氢酶［例（％）］	
>250 U/L	10（66.7）
≤250 U/L	5（33.3）
巨大包块［例（％）］	
有	4（26.7）
无	11（73.3）
移植前接受二线及以上治疗［例（％）］	
是	2（13.3）
否	13（86.7）
移植前疾病状态［例（％）］	
完全缓解	10（66.7）
部分缓解	2（13.3）
稳定/进展	3（20.0）

2. 造血重建情况：移植时回输的造血干细胞采集物CD34^+^细胞中位数为6.72（2.47～14.45）×10^6^/kg。15例患者中位粒细胞植入时间为移植后第12（10～18）天，中位血小板植入时间为移植后第12（10～19）天。移植期间输注滤除白细胞悬浮红细胞中位数为2（0～7）个单位，输注滤除白细胞机采血小板中位数为3（3～7）个单位。

3. 不良反应：移植100 d内TRM为0。8例患者出现3～4级发热性中性粒细胞减少。10例患者出现3～4级贫血。常见的非血液学不良反应为胃肠道症状，但3级和4级不良反应少见，其中发生3～4级恶心/呕吐、腹泻均为2例。口腔黏膜炎（5例）和呼吸道感染（4例）也是较为常见的不良反应，而发生肝功能异常、急性肾损伤、心律失常者不常见（表2）。

**表2:** 15例接受含苯达莫司汀预处理自体造血干细胞移植恶性淋巴瘤患者的不良反应［例（％）］

不良反应	1~2级	3~4级
发热性中性粒细胞减少		8（53.3）
贫血	5（33.3）	10（66.7）
胃肠道不良反应		
腹泻	3（20.0）	2（13.3）
恶心/呕吐	5（33.3）	2（13.3）
肝功能异常		
转氨酶升高	1（6.7）	1（6.7）
ALP升高	0（0）	1（6.7）
高胆红素血症	1（6.7）	0（0）
感染		
呼吸道感染	0（0）	4（26.7）
口腔黏膜炎	5（33.3）	1（6.7）
急性肾损伤	1（6.7）	0（0）
心律失常	1（6.7）	0（0）

4. 疗效分析：移植后3个月评估疗效，12例患者获得CR，1例患者获得PR，2例患者评估为PD。ASCT前获得CR的7例初治患者在移植后仍为CR。8例复发难治患者，在移植后5例获得CR，其中3例在移植前即通过挽救治疗获得CR，1例获得PR，1例为PD。2例ASCT后评估为PD患者在移植前分别处于PR、SD状态。另1例ASCT前评估为PD患者在移植后获得PR。

## 讨论

ASCT适用于对化疗敏感、年龄较轻且体能状态较好的具有不良预后因素的非霍奇金淋巴瘤（NHL）的一线诱导化疗后的巩固治疗；也适用于一线治疗失败后挽救化疗敏感患者的巩固治疗[9]。国内缺乏含Ben预处理方案的临床数据。因此，本研究回顾性分析了接受BeEAM或者BeEAC作为预处理方案的15例恶性淋巴瘤患者的近期疗效及安全性。

Visani等[10]–[11]初步报道了BeEAM方案在复发/难治性淋巴瘤患者中的疗效，中位随访18个月，81％的患者获CR。NHL患者进行BEB方案（白消安、依托泊苷和Ben）预处理时，移植后3个月的CR率为81.8％[12]。本研究中，移植后3个月CR率为80％，与上述研究疗效相当。ASCT后获得CR的12例患者，包括ASCT前即获得CR的7例初治患者、4例经挽救治疗获得PR/CR的复发难治患者，仅1例为移植前疗效评估PD的复发难治患者。ASCT后3个月内2例患者发生PD，其中1例为滤泡淋巴瘤转化为弥漫大B细胞淋巴瘤，1例为血管免疫母细胞性T细胞淋巴瘤，移植前接受二线或三线治疗，移植前疗效评估分别为PR、SD。上述结果提示ASCT前疾病缓解状态可能影响了疾病的疗效[11]。由此可见，含Ben预处理方案联合ASCT为治疗初治高危、复发难治淋巴瘤的有效方案。

复发/难治淋巴瘤患者进行BeEAM方案预处理时，粒细胞植入的中位时间为第10天，血小板植入的中位时间为第13天[10]。本研究纳入15例淋巴瘤患者，接受BeEAM或者BeEAC方案预处理，与以往含Ben预处理方案相比，植入时间无明显差异。含Ben预处理方案，最常见的3～4级非血液学不良反应包括黏膜炎、消化道症状、发热、败血症和急性肾脏衰竭等[13]–[15]。在LYSA和GELTAMO研究组报道的BeEAM预处理方案中，急性肾损伤的发生率高达23.3％～27.9％[16]–[17]；多变量分析时，移植前慢性肾功能衰竭、Ben剂量>160 mg/m²和年龄>55岁是急性肾功能衰竭的独立预后因素[16]。而本研究中较低的肾功能损伤发生率可能与Ben剂量减低有关。同时，本研究观察到其他2级以上不良反应如发热性中性粒细胞减少、口腔黏膜炎、呼吸道感染等，与既往报道的含Ben预处理方案相比无显著差异[10],[13],[16]，且较容易得到控制。

本研究结果表明，以BeEAM或BeEAC方案作为淋巴瘤ASCT的预处理方案是安全有效的。然而，本研究存在入组病例数少、未能行剂量范围探索、随访时间短、无法对远期疗效进行分析等局限性，后续我们将进行多中心、前瞻性、随机对照研究，进一步评估含Ben预处理方案的优劣。
